# Mentoring in STEM higher education: a synthesis of the literature to (re)present the excluded women of color

**DOI:** 10.1186/s40594-022-00367-7

**Published:** 2022-07-29

**Authors:** Tara Nkrumah, Kimberly A. Scott

**Affiliations:** Center for Gender Equity in Science and Technology, Arizona State University, Community Services Building, Room 361A, 200 E. Curry Road, Tempe, AZ 85281 USA

**Keywords:** Higher education, Intersectionality, Literature review, Mentoring, STEM, Women of color

## Abstract

Mentoring initiatives for undergraduate and graduate women of color (WOC) have provided peer-to-peer relationships and counterspaces to disrupt the inequitable treatment of students in STEM higher education (HE). This literature synthesis explores intersectionality in STEM HE mentoring through pursuing the following research questions: (1) What impact do the social contexts of WOC have on their mentoring experiences in STEM HE? (2) What role does intersectionality play in the structural organization of WOC mentoring models in STEM HE? (3) How has intersectionality shaped the life experiences of WOC mentors and mentees? and (4) How can mentoring models utilize intersectionality to incorporate the experiences of WOC in STEM HE? Thematic findings from literature related to STEM HE mentoring suggest a reinforcement of deficit mentoring models (Fix the URM), a symbolic application of intersectionality (branding gender–race), and a lack of paradigmatic shifts (catch-all). Our specific recommendations presented in this article challenge the epistemic oppression and epistemic violence that current STEM HE mentoring models operationalize.

**Supplementary Information:**

The online version contains supplementary material available at 10.1186/s40594-022-00367-7.

## Introduction

Women of color (Black/African American, Hispanic/Latina^1^, and American Indian/Alaska Native/Native American^2^, Pacific Islander) undergraduate and graduate students are very underrepresented in STEM higher education (HE) departments. Some of the purported reasons for this phenomenon include: (1) STEM programs nurture an environment that excludes women of color (WOC) (Smith et al., 2019); (2) HE’s misunderstanding of and/or lack of interest in the unique barriers and experiences faced by women of color (Guy & Boards, 2019); and (3) postsecondary institutions’ assumptions that all women of color lack proper academic preparation to succeed in STEM courses (Ghee et al., 2016). In short, the culture of many STEM departments and the organizations in which they function create structures reifying White maleness at the expense of women of color (Clancy & Davis, 2019; Dancy et al., 2020; Johnson, 2011). Consequently, WOC frequently encounter forms of race–ethnic–gender discrimination and oppression (Espinosa, 2011; Perna et al., 2010). Although women of color have created multiple forms of resistance to inequity in STEM HE, including establishing peer-to-peer relationships and counterspaces—namely, those “safe spaces” that “lie in the margins, outside of mainstream educational spaces, and are occupied by members of nontraditional groups” (Ong et al., 2018, p. 206)—the potential to disrupt the traditional power structures that are prevalent in the cultures of STEM HE needs to be systematically examined. To that end, intersectionality, a critical framework that encourages analysis and active interrogation of interconnected oppressive structures, is well suited for this work. Collins (2019) characterizes intersectionality as a “metaphor”, and encourages scholars and practitioners to explore “the connectedness of different systems of power”, and within the analysis, the significance of “theorizing power relations and political identities” is recognized (p. 30). We understand intersectionality as far more than multiple identity theory. Stated differently, an intersectional approach recognizes individuals possess a multitude of characteristics (e.g., race, gender, sexuality, ethnicity, ability and so on) *and* necessitates a more nuanced framework to exploring WOC’s STEM experiences.

Ireland, Freeman, Winston-Proctor, DeLaine, Lowe, and Woodson (2018) have argued that “a greater diversity of researchers and research approaches are needed to operationalize intersectional experiences of Black girls [and girls of color] and women in STEM” (p. 246). Our article responds to the call of Ong et al. (2018) for examining the “differences among women of color from varying racial/ethnic backgrounds and STEM fields” (p. 237). This project focuses on specific race–ethnic–gender groups, recognizing that there are additional identity markers which are just as important (e.g., sexuality, immigration status, language, etc.) but are not examined herein due to space. We analyze how presumed liberatory efforts (e.g., mentoring models for women of color in STEM) within an infrastructure (e.g., HE) reinforce matrices of power and domination, further subjugating WOC in STEM. Three questions shape this intersectional examination: (1) What impact do the social contexts of WOC have on their mentoring experiences in STEM HE? (2) What role does intersectionality play in the structural organization of WOC mentoring models in STEM HE; (3) How has intersectionality shaped WOC mentors’ and mentees’ life experiences? and (4) How can mentoring models utilize intersectionality to incorporate the experiences of WOC in STEM HE?

To answer these questions, we must first contextualize race–ethnicity–gender data for WOC STEM students in HE. This first section illustrates how clear disparities indicate systemic issues despite a history of mentoring. The ways in which mentoring approaches have been positioned in general, and with underrepresented groups in STEM in particular, point to the need for a more nuanced analysis of social theory. Consequently, the second section of this paper explicates intersectionality as a theoretical framing of “critical social theory in the making” (Collins, 2019, p. 23) to unpack complex hierarchical structures. The third section presents how mentoring and intersectionality led to our four research questions. The subsequent Methods section explains how we winnowed more than 20,000 peer-reviewed articles down to the 45 that were analyzed for this synthesis. After the fourth section reveals the three primary themes from our analysis, we offer a discussion of the findings in the fifth section, where we provide specific recommendations. The Conclusion describes how our analysis can be used given the tenor of the times.

## Background

### Women of color in STEM

While there has been an increase in STEM programs aimed at serving WOC in HE (Aikens et al., 2017; Eubanks-Turner et al., 2018; Smith & Wingate, 2016), statistical data fail to show significant evidence of progress made to end or ease the disparities these women face in HE. Table 1, which is from the 2019 “Women, Minorities, and Persons with Disabilities in Science and Engineering” report, includes race/ethnic gender percentages in bachelor’s degrees conferred by discipline to US citizens and permanent residents. For all STEM degrees combined, White women represent 34.5%, Black women 6.3%, Hispanic/Latina women 7.0%, and Asian women 2.6%. As can be seen, the pattern was very similar, but lower in absolute value for women of color in the other STEM disciplines. Overall, the percentage of women earning degrees was low, in addition to the fact that women of color have appreciably lower representation than White women in all STEM disciplines.

**Table 1:** Percentage of degrees conferred by STEM discipline for women (2016)

Participation by %	White women	Black/African American women	Hispanic/Latina women	Asian women
Population	34.5	6.3	7.0	2.6
BioSciences	54.8	4.5	7.04	7
CompScience	18.7	2.2	1.87	< 1
Math/Stats	42.4	2.1	3.73	< 1
Engineering	20.9	1.0	2.31	< 1
PhysicalScience	19.3	2.5	3.73	< 1

Adapted from “2019 women, minorities, and persons with disabilities in science and engineering report”, by the National Science Foundation, National Center for Science and Engineering Statistics. 2019. *Women, Minorities, and Persons with Disabilities in Science and Engineering: 2019.* Special Report NSF *19*–304. Alexandria, VA. (https://www.nsf.gov/statistics/wmpd). Copyright 2019 by NCSES

**Fig. 1:**
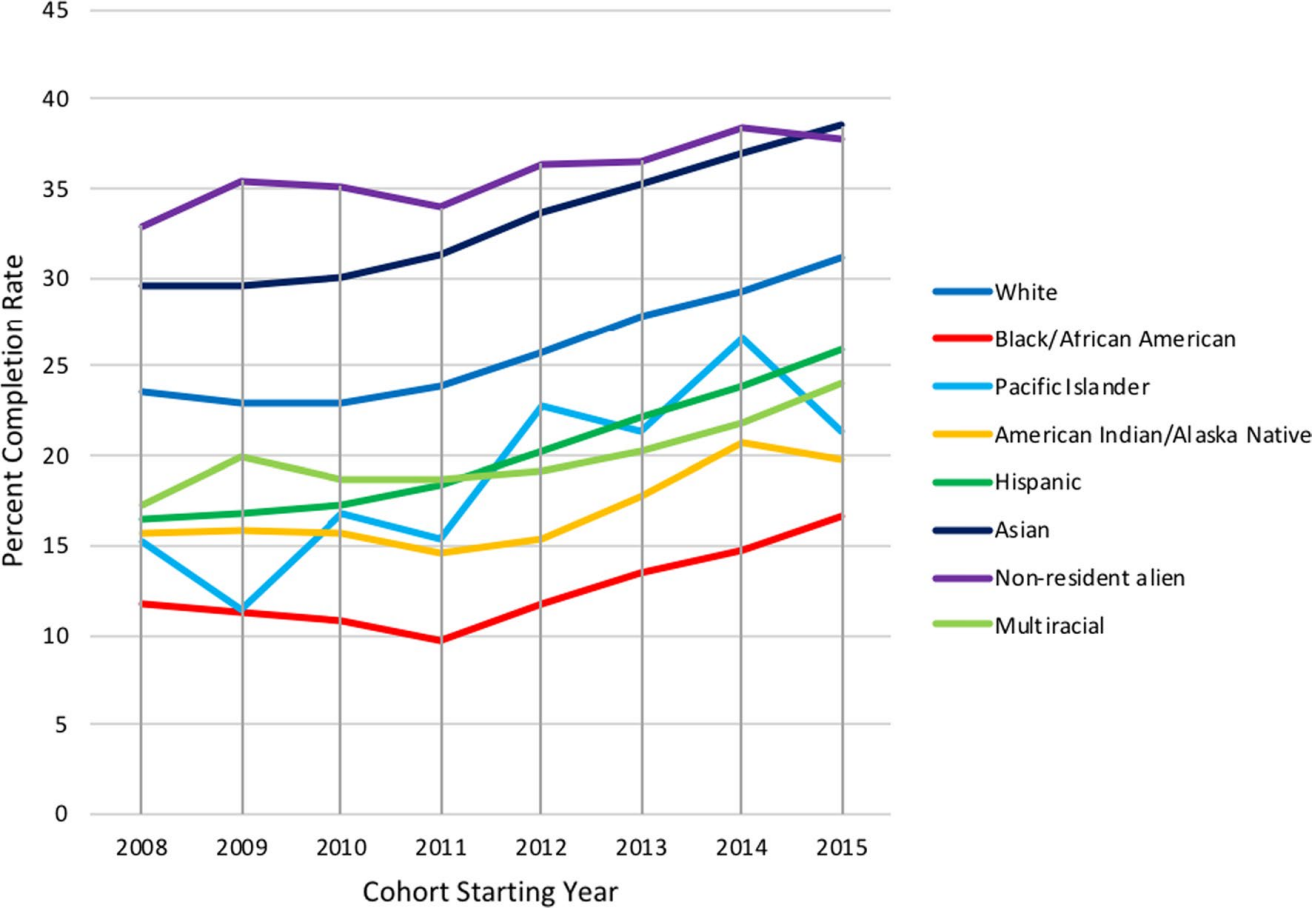
NCES/IPEDS—graduation/completion rates over time. 2008 was the first year Asian and Pacific Islanders were disaggregated and results from Multiracial category (“Two or more races”) were reported

Placing the graduation/completion rates for bachelor’s degrees earned by the percentage of ethnicity/gender in each degree field (2016) out of all bachelor’s degree earned over time for first-time full-time students at a public institution (Fig. 1), it is possible to track the completion rate for a cohort of students from 2008 to 2015.^3^ This calculation reveals that Black/African American female students had the lowest percent completion rates, with the cohort of 2008 graduating almost 12% of students and the cohort of 2015 graduating nearly 17%. While growth has occurred in the percent completion rates of Black/African American female students, it is far behind the completion rates of other groups. The most erratic trend line is that of Pacific Islander students, with their 2008 cohort graduating just over 15% of students, only to fall to 11.4% the next year (2009), before jumping up to 16.8% in 2010. Once again, while there is positive growth in the completion rate for these students (21.3% completion rate for the 2015 starting cohort), it still trails other student groups, including White and Hispanic women, and may continue to jump and fall erratically.

The above trend lines provide visual proof of the existing unyielding gaps between the success rates of White women and WOC in the U.S. STEM HE system. The disaggregation by race and ethnicity for WOC shows cohort completion percentages that lag far behind White women every year. These rates, when combined with the degree participation percentages of WOC, illustrate the inability of existing STEM HE systems to serve women of color in a meaningful capacity. In short, these data are symptomatic of deeper structural problems. Importantly, the gaps should not be interpreted as pointing to the superiority or more inherent grit of certain groups. Indeed, research suggests multiple reasons for these disparities, such as a lack of access to the technological, financial, and cultural resources that are required to navigate normative HE systems (Brayboy et al., 2012; Shotton et al., 2013; Waterman, 2019; Winterer et al., 2020); hostile STEM climates impacting recruitment and persistence rates (DiBartolo et al., 2016; Kendricks et al., 2013; Leath & Chavous, 2018; Mondisa, 2018; Russell et al., 2018); social isolation and the psychological impacts of incivility and harassment (Rodrigues et al., 2021); and a system lacking proper supports for WOC to persist in STEM HE (Mondisa, 2018). This last explanation is the focus of our paper.

### Brief history of mentoring

Although the concept of mentoring has formally existed in some form since the early days of Greek and Roman education (Colley, 2002; Osipov et al., 2019; Roberts, 2000), its use in modern education systems can be traced to the early 1900s. Irby and Boswell (2016) observed how conversations around the term mentoring transitioned from social settings into school cultures (i.e., the founding of Junior Achievement). Over time, educational research has advanced expectations of mentoring to produce favorable outcomes (see Figueroa & Rodriguez, 2015; Luedke, 2017). Despite being prevalent in STEM HE, the term mentoring renders multiple definitions, such as meaningful interactions between individuals (McCoy et al., 2015; Mondisa, 2015) and program implementations, including faculty mentoring or apprenticeship models (Amelink, 2008; Luedke, 2017).

In 2008, Amelink published an overview of mentoring that described the positive socialization process for women in STEM, specifically in engineering. Amelink’s extensive overview featured nine models of mentoring with brief descriptions of each. Defining mentoring as meaningful interactions between highly skilled and a lesser skilled people, Amelink’s research acknowledged the theoretical and conceptual complexity of STEM mentoring to enact approaches that produce equity at the systemic level in STEM education/careers. However, her research did not account for the unique experiences of sociohistorically disenfranchised student populations—that is, WOC. As a consequence, Amelink’s mentoring models assume a one-size-fits-all mentorship approach (McCoy et al., 2015), ignoring core principles that a more nuanced approach would center. Stated differently, without considering relationality, the interconnections between social categories, subject formations, and power structures, mentoring becomes a means to adopt coping strategies (see Morganson et al., 2010). Such approaches do little to recognize, analyze, and/or dismantle the institutionalized factors reinforcing a system of dominance and subordination.

Many mentoring models with an explicit aim to support “underrepresented students” in HE focus on “upskilling” students through supplemental academic instruction (Slovacek et al., 2011; Wilson et al., 2012). Understanding that many of these students lack appropriate high school resources (e.g., qualified classroom teachers), HE mentors intervene by providing laboratory experiences and structured research opportunities (Haeger & Fresquez, 2016; Schneider et al., 2015). Even without disaggregating the data along race and gender divides, the results of such approaches’ effectiveness are varied. For instance, Lisberg and Woods’s (2018) integrative study examining a STEM bootcamp approach to retain underrepresented minority (URM) students majoring in STEM reported successful outcomes, reaching higher retention rates in the first 2 years; nonetheless, retention rates dropped in the third year. Despite the academic support that required meetings with peer mentors, faculty mentors, and STEM faculty of enrolled STEM courses, the underrepresented students still showed lower retention in comparison to non-underrepresented students in the third year of STEM programs, at 65.6% and 71.5%, respectively.

Questions linger as to what occurs *while* programs try to improve persistence and success for WOC in STEM HE (Mondisa, 2018). More recently, intersectionality has been used as a tool to examine the outcomes of problematic STEM contexts that WOC endure, through studies of STEM camps targeting academic skills (Lisberg & Woods, 2018) or learning communities that closely monitor student achievement in mandatory monthly meetings (Scott et al., 2017). For example, Smith et al. (2019) describe Black women’s overcoming attitudes in STEM as possessing “high private regard”. When women express feelings of marginalization, Smith et al. label these sentiments as examples of “low public regard”. The degree to which Black women operationalize their “high private” or “low public” regard affects their STEM success. These studies lay the foundation for understanding how mentorship for other WOC in STEM HE can be improved. Given demographic changes, understanding what is not working in current STEM HE mentorship models is crucial.

In 2017, WOC were the fastest-growing population in the United States, constituting nearly 21% of the total population and 41% of the total female population, with estimated projections putting them at 30% of the total population and nearly 60% of the total female population by 2060 (U.S. Bureau of the Census, 2017). These women will enter the workforce when nearly all the fastest-growing jobs in the United States are within STEM professions, and around 6% of the total population is employed in STEM-based occupations (U.S. Bureau of the Census, 2019). To keep pace with the rapid growth in the STEM workforce, employers must hire the nation’s fastest-growing employable groups—WOC (U.S. Bureau of the Census, 2019; Ahmad & Iverson, 2013). The inclusion of WOC will add unique perspectives and innovative ideas currently absent from the STEM workforce. Given these statistics, WOC STEM students need different support than what is presently provided. A more nuanced analysis of mentoring strategies is needed to determine how the support should be offered. An intersectional analysis has significant potential because its “goals of social reform and social transformation influence the critical discourse that arises in order to move toward those goals” (Collins, 2019, p. 82).

In seeking to answer our questions, we follow Collins (2019) and her call to “sharpen intersectionality’s critical edge” through an exploration of relationality, power, and social processes (p. 226). We hold HE as a “saturated site of power” (Collins, 2019, p. 235). The next sections outline how we envision intersectionality as a tool for analyzing the social forces working across the practices, patterns, and representations of everyday social interaction in HE (Collins, 2019). We begin with a critical definition of intersectionality and some of its key concepts before moving on to its utilization in our current project.

### Theoretical framing

Kimberlé Crenshaw (1989) introduced the term intersectionality to legal studies to describe “double discrimination” against Black women, or “the combined effects of practices which discriminate on the basis of race, and on the basis of sex” (p. 149). Although many researchers cite Crenshaw’s scholarship as the origin of intersectionality research, intersectional inquiry and praxis predate the coining of the term.

Intersectionality has a historical legacy as a critical social theory, topic for debate, and/or methodology. For instance, critical feminists, including Sojourner Truth, Anna Julia Cooper, the Combahee River Collective, Audre Lorde, bell hooks, Gloria Anzaldúa, and Patricia Hill Collins, have used intersectionality as an analytical framework to describe, analyze, and catalyze social justice movements among WOC (see, for example, Anzaldúa, 1987; Collins & Bilge, 2016; Collins, 2019; Combahee River Collective, 1983; Guy-Sheftall, 2009; Keating, 2006; Martinez, 2002). Other scholars have debated how well intersectionality can be used to theorize multiple forms of oppressions (Ferguson, 2012; McCall, 2005) and how easily it can be put into practice (see Berger & Guidroz, 2009; Dill & Zambrana, 2009; Harris & Patton, 2019; McCall, 2005; Puar, 2012; Shields, 2008). Other studies present its impact on the research process and the forming of research questions (Collins & Bilge, 2016; Puar, 2012; Shields, 2008). More recently, Collins (2019) presents intersectionality as an analytic strategy, methodological tool, and form of critical praxis and inquiry (Collins, 2019).

In this paper, we combine Crenshaw’s perspective on intersectionality as a strategy with Collins’ description of it as an analytical tool. For our purposes, we use intersectionality as a frame to assist us in describing current HE mentoring for WOC in STEM. We further utilize intersectionality as a strategy to expand mentoring efforts to be more socially just. Intersectionality allows us to critically describe what conditions exist currently and envision an equitable future. Together, we recognize that intersectionality is organized around a set of core constructs: social context, relationality, power, social inequality, complexity, and social justice (ibid). Given this complexity, we introduce each construct to describe what it signifies; how it is generally used; and how we apply it to this work.

## The application of intersectionality to mentoring women of color in the STEM HE

### Social context

What: An intersectional frame considers the social context, which is where, when, and with whom knowledge production occurs. Thus, a social context is more than the physical locale in which individuals find themselves. It concerns how an individual interprets the context and how the context influences an individual’s intersecting identity markers (e.g., race, gender, ethnicity, sexuality). How: Attention paid to the social context can highlight the perspectives, histories, politics, and communities that influence individual and group identities. Through having a heightened sensitivity to such constructs, we see that there are many historical, interconnected layers contributing to the present-day situation of marginalized groups (Anzaldua & Keating, 2013; Collins, 2019; Dill & Zambrana, 2009). Our application: We examined how participants (e.g., WOC postsecondary STEM students) discussed in other research studies navigated specific social contexts in HE. An intersectional lens illuminates the complexity of experiences and makes the barriers visible that are hindering true progress for WOC in STEM.

### Relationality

What: Relationality refers to analyzing and understanding the connections among race, class, and gender. When adopting an intersectional lens, relationality necessitates the rejection of either/or binary thinking. Instead, a both/and framework is embraced to identify how power informs the relationships among race, class, gender, and other identity markers. Stated differently, our African American femaleness cannot be captured by simply saying we are women. And in some social contexts, our sexuality combined with our Black femaleness will endow us with certain power while in other social contexts these same markers will disempower us. How: People understand their social positions through relational or interconnected processes of association (Anzaldúa, 1987; Collins, 2019; Collins & Bilge, 2016). Understanding how intersecting components surface during individuals’ interactions will expose the “social positions occupied by actors, systems, and political/economic structural arrangements necessarily acquiring meaning and power (or a lack thereof) in relation to other social positions” (Collins, 2019, p. 46). Our application: In mentoring models, relationality emphasizes the ways mentoring gains meaning through interactions between mentors and mentees. This study characterizes the processes of socialization in STEM HE disciplines, thereby affecting mentees’ fluid, interconnected social positions.

### Power

What: Intersectionality recognizes that *power* operates across and through interpersonal, disciplinary, cultural (hegemonic), and structural domains (Collins, 2019; Collins & Bilge, 2016; Dill & Zambrana, 2009). Power is mutually constitutive and constructed. It is co-created through interactions. It is through relationships that power appears. Stated differently, power can be picked up or given as individuals interact with each other and within particular social contexts. Like other poststructural feminists, we define power as a set of forces that is “everywhere, not because it embraces everything, but because it comes from everywhere” (Foucault, 1978, p. 93). How: Power manifests itself in multiple forms yet is often so invisible that researchers miss its potency and pervasiveness. An intersectional lens uncovers how power influences relationships and structures across multiple domains—such as interpersonal, disciplinary, cultural, and structural domains (Collins, 2019; Collins & Bilge, 2016; Dill & Zambrana, 2009). Power is a resource that can be used to understand the sources of inequity and unequal distribution of other resources. By recognizing how power connects through these domains, we come to understand how race–ethnic–gender hierarchies maintain themselves. Our application: In mentoring, power operates through multiple domains at once. Nevertheless, the interpersonal, structural, and disciplinary domains spotlight individual mentoring relations and processes, the academic setting, and the reward systems within specific social contexts. Unsurprisingly, power structures lead to social inequality.

### Social inequality

What: We understand *social inequality* as the material and social realities of inequity that result from the oppression and domination of one social group by another. By using an intersectional lens, researchers examine and actively resist widespread assumptions about natural inequalities between people based on their membership in race and gender groups (e.g., WOC are not motivated to enter or persist in STEM). How: By building knowledge and socially just actions, social inequality can be both interrogated and transformed. An intersectional framework spotlights that inequality is not “natural” or “inevitable”, despite its being normalized (Collins, 2019). Our application: Mentoring that focuses on socializing students into dominant STEM HE norms ignores the patterns of *social inequality* that necessitate such approaches. We recognize such approaches as assimilatory, assuming an a priori deficit model. Critiques of the structures that reinforce this paradigm are conspicuously absent. Consequently, social inequality persists, due in part to its invisibility.

### Complexity

What: Complexity refers to social inequality, power, relationality, and social contexts as intertwined, fluid, and malleable. While these constructs operate on many levels simultaneously, one cannot be understood without the other. How: When used together, these constructs *“*give people better access to the complexity of the world and of themselves” (Collins & Bilge, 2016, p. 2). Simple approaches to examining issues of power cannot be accurate given the complex nature of the systems. Although a single unit of analysis tends to be more widely accepted than using an intersectional approach, this does not mean it is better. Stated differently, using the term “women” as the only unit of analysis will not capture the totality of all women’s experiences. Additionally, the term “women” tends to center White womanhood. Our application: We recognize that variations exist, even within certain populations*.* In mentoring relationships, studying a category such as underrepresented students or WOC with an intersectional lens requires a consideration of the complexities and variations within and across populations (i.e., class, nationality, ethnicity, geographic location, ability, sexuality, gender identity, etc.). This move toward critically analyzing intra- and intergroup differences is one step toward socially just action.

### Social justice

What: Social justice is action oriented. Through an intersectional lens, social justice is a tool that can be used to effectively confront and transform inequity. How: When intersectionality is used to help students learn how to deconstruct inequitable HE systems, the needs and experiences of WOC can be appropriately centered. The intersectional frame positions social justice as a means through which systemic transformation can occur. Transformation requires more than adding new programs or efforts to a flawed system. Instead, as an intersectional core construct, social justice requires careful exploration of how the system remains unjust; who benefits and remains disenfranchised by the injustice—such as WOC; and how and where subordinated groups have transformed and could transform the system towards more equitable ends. Our application: Although some mentoring models provide a mix of traditional and culturally based strategies for addressing underrepresentation (Louie & Wilson-Ahlstrom, 2018; Mondisa, 2015, 2018; Perna et al., 2010; Slovacek et al., 2011), unpacking the ways these efforts lead to social change remains missing. We assume an ethical motivation and commitment of intersectional scholarship to maintain a critique of the status quo and dominant operations of power. Drawing on Collins (2019), we aim to effect change in the research process through activist praxis and scholarly knowledge production.

Collectively, the above constructs influence the four research questions directing this study (see Table 2). We also applied intersectionality as a methodological tool, which is discussed further in the next section.

**Table 2:** Intersectionality mentorship framework

Core construct	Definition	Construct within mentoring context	Research question
Social context	Locations or nexuses of identities, in which individuals and systems interpret each other along varying intersecting lines (e.g., race, gender, ethnicity)	Contextualizing mentoring relations within HE systems	1) What impact do the social contexts of WOC have on their mentoring experiences in STEM HE?
Relationality	Interconnected and mutually productive contextualized relations among social categories	Mentoring gains meaning through the relational interactions between individuals	
Power	Interconnected, mutually productive systems and structures of domination and oppression	Mentor–mentee relationship creates a power dynamic reinforced, or not, by situated systems and structures	2) What role does intersectionality play in the structural organization of WOC mentoring models in STEM HE? 3) How has intersectionality shaped the life experiences of WOC mentors and mentees?
Social inequality	Material and experiential outcomes that are the result of socially constructed relations depicted as natural	Mentoring that ignores the patterns of social inequality	
Complexity	Social inequality, power, relationality, and social context are intertwined	Variations within certain populations require intragroup, contextualized examinations, and practices of mentoring	
Social justice	Center social transformational change and holistic interactions in activist praxis and scholarly knowledge production	Mentoring can lead to deconstructing and transforming inequitable HE systems	4) How can mentoring models utilize intersectionality to incorporate the experiences of WOC in STEM HE?

## Methodology

Our review of the literature on STEM mentoring for WOC in HE was done in the following three phases: (1) searching for peer-reviewed empirical articles, (2) abstract screening, and (3) full article screening. We examined the state of the literature from three databases: Academic Search Premier, ERIC, and PsycInfo. We situated the literature search in the geographical area of the United States. Although the U.S.-based articles were from geographically diverse regions, most came from Southwestern states. The selection of keywords was intentional. We selected STEM, mentor, and intersectionality as keywords to explore the strategies used to address the ongoing underrepresentation by gender and race in STEM education. Typical models follow standardized approaches (e.g., content knowledge development) based on dominant norms to mentor individuals from multicultural and linguistic communities. Such shortsighted techniques inform the research questions to examine for the influence of variables (i.e., social contexts, structural organization, lived experiences) on STEM mentorship for WOC. For instance, the intersectional construct of context is important for revealing specific saturated sites of violence. Consequently, we used the keywords “higher education” in order to explore this space. The keyword “STEM” in this study is limited in scope and reflects the mainstream reference to the four categories of science, technology, engineering, and mathematics to increase the likelihood of finding related articles. We also used the word “mentor*” (with the asterisk) to allow for verb forms of “mentor” to be included in our search (i.e., mentor*s*, mentor*ing*, mentor*ed*, etc.). Additionally, keyword searches included variations of “African American or Black”, “Native American and Indigenous”, “Hispanic and Latina/x”, “Asian American and Asian”, “girls or women”, “girls of color”, “women of color”, and “underrepresented”. This was purposeful because of our understanding from intersectionality that relationality encourages both/and approaches. Specifically, mentors, mentees, and mentoring are all affected by the situated meanings of race, ethnicity, and gender. Admittedly, there are intersections among groups. Exploring African American women who are differently able-bodied or Latina women who self-identify as members of the LGBTQIA + communities, for example, better reflects the heterogeneity beyond race, ethnicity, and gender. We acknowledge that WOC possess and navigate multiple identities in HE and beyond. However, we focus on their race and gender only as entry points to the process of using an intersectional framework.

Importantly, our search did not reveal any articles discussing Asian American women in regard to STEM HE mentoring programs. Despite the fact that Asian women are enrolled in higher education at lower rates (2.6%) than White women, they tend to be overrepresented in STEM disciplines (Funk & Parker, 2018). Yet, research indicates that Asian women face many obstacles and constraints in STEM HE programs (Castro & Collins, 2021) and the STEM workforce (Williams et al., 2014). We did not use the keyword “Pacific Islander” in our search. This decision was not made to infer that this group is insignificant. Indeed, Pacific Islanders are among the fastest-growing female populations in the United States (Ramakrishnan & Ahmad, 2014). Yet the vast majority of research defines underrepresentation based on the absence of African American, Latina/Hispanic, and Native American. We worked within the confines of these limitations, recognizing Pacific Islanders’ invisibility is often hidden within the Asian American category (e.g., Asian American Pacific Islanders).

Among the three databases probed, initial hits revealed 22,306 articles tagged with some combination of “STEM” + above additional keywords; 13,142 tagged with some combination of “mentor*” + above additional keywords; only 107 articles tagged with some combination of “intersectionality” + above additional keywords. We worked collaboratively and iteratively to reduce these unique sources for our project.

### Inclusion and exclusion criteria

Originally, we intended to identify empirical works that exclusively named WOC mentoring in HE. However, this approach quickly resulted in few studies. Rather than continue with this initial strategy, we discovered that the “underrepresented” category provided us with examples in which WOC appeared. Modifying our search strategy to keywords with “underrepresented groups” as the descriptor illuminated and emphasized the larger issue, which is that mentoring frameworks are not designed for WOC.

Initial searches on Academic Search Premier, ERIC, and PsycInfo revealed 35,499 total articles. We grouped the search keywords and their various permutations into three groups (“mentor focus”, “STEM/identity focus”, and “intersectionality focus”) to differentiate article totals and calculate percentages. This grouping revealed 22,306 articles tagged with some combination of “STEM” + additional keywords; 13,086 tagged with some combination of “mentor*” + additional keywords; 107 articles tagged with some combination of “intersectionality” + additional keywords. From the 35,499 total articles, we narrowed our selection to articles with “STEM”, “underrepresented”, “higher education”, and “mentor*” tagged or mentioned in the abstracts because we predicted that combination of keywords would give us a selection of articles most relevant for this study. That search resulted in 99 total articles among the three databases, 16 of which overlapped and 40 of which were eliminated because they were deemed not relevant through a close reading of their abstracts and paper materials.

If the abstract provided information based on four criteria, we moved them to full article review: (1) empirical studies whereas conceptual articles without results were excluded; (2) mentoring is centered as a significant HE strategy, meaning details the application and role of mentoring or the mentoring framework; (3) clear articulation of WOC or underrepresented women as mentees disaggregated based on race and/or ethnicity; and (4) the focus of the mentoring program was for undergraduate or graduate STEM students; mentoring faculty to be mentors was not our intent. Also, we mined the references of selected articles to identify additional sources for the literature review. If articles upheld the same standards of the target population (i.e., WOC) and were U.S.-based studies, they were included. For instance, pilot studies from a multiple-year grant (e.g., NSF 5-year award) that focused on the influence of mentoring underrepresented groups and reported outcomes were included.

The timeline for our literature review was between 1990 and 2019; we conducted electronic searches of peer-reviewed articles in relevant journals. We began our exploration in 1990 since this was when educational efforts emphasized improving engagement for students of color. Work about culturally relevant teaching rightly assumed centerstage in the 1990s (see, for example, Ladson-Billings, 1994, 1995). However, we soon discovered what Ong, Wright, Espinosa and Orfield (2011) revealed: that few works about WOC in STEM were published between 1970 and 2008.

As a result of the abstract review, 99 article sources were identified and narrowed to include 45 empirical articles. The articles were filtered based on the criteria to provide pertinent information on HE’s STEM mentoring model goals, objectives, and outcomes. The significance of narrowing the focus on mentoring in STEM HE for underrepresented groups was to explore the impact on WOC.

### Data analysis

Drawing on Miles and Huberman (1994), three phases allowed us to identify nine codes (near-peer mentor, peer mentoring, comprehensive mentoring, peer mentoring circles, faculty mentoring, hierarchical mentoring, cultural-awareness mentoring, formal mentoring, doctoral STEM mentors) among the 45 articles. Phase one included a careful exploration of the 45 articles to identify whether any of them mentioned a mentoring model. This initial reading led to the nine codes. Phase two included examining the 45 articles to categorize what types of mentoring models appeared. For instance, we noted reasons for mentoring, including encouraging interest in STEM, building content knowledge, and creating a sense of belonging. The third phase involved noting whether any of the 45 articles made references to intersectionality or its core constructs. At the completion of the three phases, themes were identified and organized into a Google spreadsheet which assisted the team in recognizing how themes intersected across which points.

### Reliability and validity

We incorporated multiple steps in the coding process to ensure the trustworthiness of our results (Korstjens & Moser, 2018; Shenton, 2004). After the initial vetting process to identify the 45 articles, two members of the research team (Nkrumah & McInnes) independently conducted a close read, recording notes on a Google spreadsheet that included significant themes interpreted by the reviewer of the article. Then, we discussed each article and offered justifications for our findings. During our joint sessions, we created a new Google spreadsheet of our collective findings to assess the interpretations for consistency. In cases where our analyses of an article differed, we included it in the list to be reread and discussed. We did at least two rounds of reading the 45 articles, followed by discussions on the content (i.e., target audience, methods, findings) before confirming the themes.

From the first iteration of coding, nine different codes of mentor models were discussed. After further review, these nine mentor model types were organized under three codes determined by their assigned role and purpose in the study. The represented categories for the three codes were: faculty mentor (i.e., inclusive of formal mentoring, doctoral STEM mentors, hierarchical mentoring model), near-peer mentor (i.e., inclusive of peer mentoring circles, comprehensive mentoring), and peer mentoring (i.e., inclusive of peer-to-peer mentoring, cultural-awareness mentoring, mentoring relationships).

**Fig. 2:**
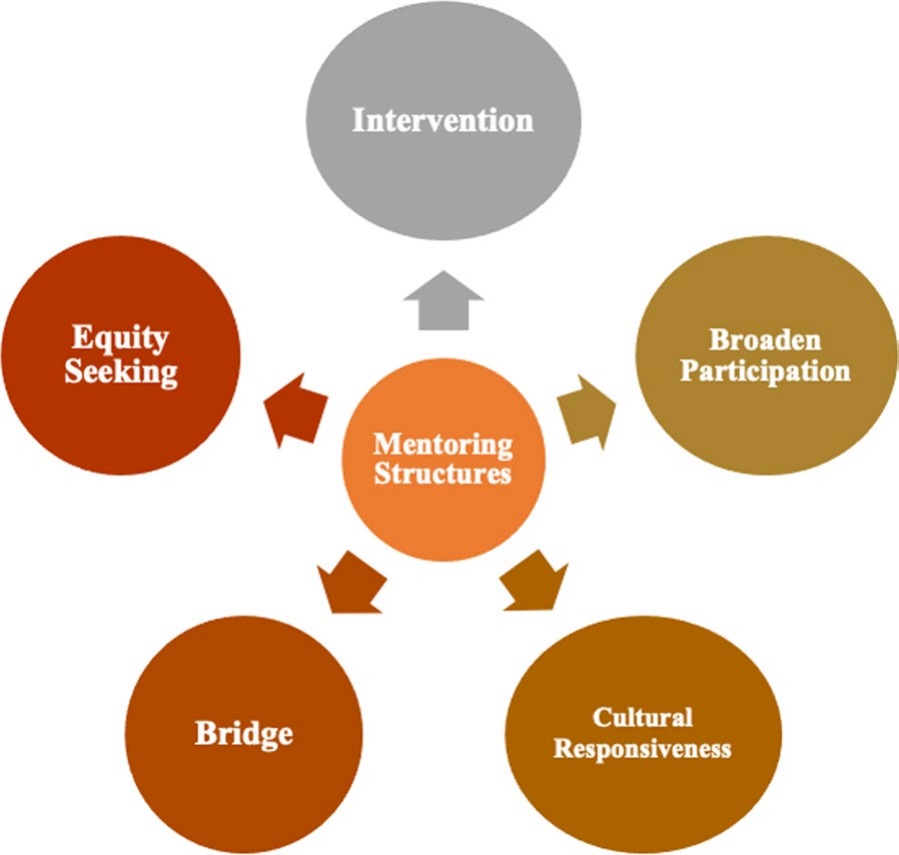
The five mentoring structures in STEM HE that represent the reason for mentoring

The second iteration of coding included examples reflective of the motivation for mentoring. We identified the following five reasons for inclusion by action-oriented agendas: (1) intervention—the creation of STEM programs for minoritized students to improve their success rate; (2) broaden participation—providing the necessary resources to overcome hindrances to student engagement in STEM education; (3) bridge—the formation of an inclusive learning environment in STEM education; (4) cultural responsiveness—a student-centered approach to learning that acknowledged other ways of knowing and being in STEM education; and (5) equity-seeking—dismantling perceived barriers to underrepresented minorities’ educational and career opportunities in STEM education (see Fig. 2).

For the third iteration of coding, we adopted the six core constructs for intersectionality provided by Collins (2019). This initial coding process resulted in the organization of articles according to how they aligned with the construct definitions illustrated in Table 2. In our reading of the 45 articles, the constructs of *social context*, *relationality*, and *power* appeared more often than the constructs of *social inequality*, *complexity*, and *social justice*. Thus, these constructs guided our analysis and discussion.

Further, we considered the research focus on mentoring participants from the targeted underrepresented groups (i.e., women of color) in STEM HE and how the research contextualizes the needs of that population. We also examined the strategies applied to implementing mentoring specific to WOC in STEM HE and the outcomes of those efforts. Where mentoring in STEM HE did not disaggregate by race or gender but included the underrepresented as a criterion, we looked for evidence of diverse methods to accommodate the demographic diversity.

### Findings: themes within the literature on mentoring HE women of color

Our exploration of how mentoring models for undergraduate and graduate WOC in STEM applying core constructs of intersectionality revealed surprises and confirmed our suspicions. Altogether, the referenced authors in this next section present empirical data on the results of mentoring WOC. The majority of reviewed work describes mentoring models to cope with social disparities (e.g., racial, gender, class) that define the culture of STEM HE (Griffin et al., 2010; Hayes & Bigler, 2013; Kendricks et al., 2013; McCoy et al., 2015; Ong et al., 2018; Tenenbaum et al., 2014; Zaniewski & Reinholz, 2016). Most importantly, we did not identify any studies that encourage mentors or mentees to explore the processes by which the disparities persist. Stated differently, and using an intersectional lens, we did not find evidence of a mentoring model in which participants engaged in exploring the four domains of power (interpersonal, disciplinary, cultural/hegemonic, and structural) constituting what Collins (2000) called the “matrix of domination” or how “intersecting oppressions are organized” (p. 18). Among the 45 empirical articles reviewed, 47% focused on gender (see Hayes & Bigler, 2013; Holland et al., 2012; Thomas et al., 2015) and 95% focused on race (see Kendricks et al., 2013; Luedke et al., 2019; MacPhee et al., 2013; McCoy et al., 2015), while less than 20% incorporated socioeconomic status (see MacPhee et al., 2013; Trujillo et al., 2015; Wilson et al., 2012) and only 6% pursued class-related issues in STEM (see Trujillo et al., 2015). Common trends in STEM HE studies that applied mentoring models prioritize identity traits based on race or gender. Only five studies of the 45 reviewed (Goonewardene et al., 2016; MacPhee et al., 2013; Schneider et al., 2015; Trujillo et al., 2015; Wilson et al., 2012) provided a comprehensive mentoring framework for underrepresented students in STEM HE. Along with the commonly discussed inequities related to race and gender, these studies added socioeconomic and class variables through qualitative and quantitative research methods.

Few studies applied intersectionality in any meaningful way. However, we noted that the last decade showed an uptick in research applying an intersectional framework when conducting studies on WOC in STEM (see Ireland et al., 2018; Ong et al., 2018; Smith et al., 2019). Nevertheless, these works did not center mentoring as the analyzed social context. Indeed, we found it challenging to create a robust list of empirical studies that exclusively focus on any one group of WOC (e.g., African American, Latina, Native American). The identified themes relate to the research questions’ emphases on factors that influence mentoring WOC in STEM along three distinct variables—social context, structural organization, and intersectionality as an analytical lens. In part, these themes articulate the variables’ roles in framing the inquiry to explore how they uniquely influence methods to mentor WOC in STEM. The themes we noted are a result of expanding our search strategies to include “underrepresented minorities”, within which the mentoring experiences of WOC appeared. Substituting this phrase permitted us to identify and analyze empirical research studies about mentoring that mentioned WOC in the aggregate or disaggregate. As a result of this shift, we identified three interconnected themes: (1) catch-all approaches that demonstrate a lack of encouraging paradigmatic shifts; (2) fix the URM efforts that reinforce deficit mentoring models; and (3) branding race–gender as symbolic applications of intersectionality.

### Catch-all approach

Mentoring models for WOC originate from a monolithic paradigm. To this point, mentoring becomes a generic treatment with no consideration of such underlying conditions as unequal power dynamics, racism, and sexism (Brown et al., 2018; Ryan et al., 2014). Granted, some results disaggregate outcomes along race–gender (see Aikens et al., 2017); however, this differentiation is secondary. Furthermore, we did not find examples of mentoring strategies that considered the social locations of such groups before implementation of a mentor program. The term “underrepresented” is used as a catch-all nomenclature to represent all non-White, non-dominant individuals. Despite the fact that important critiques have been made against the phrase “underrepresented minority” (Bensimon, 2016; Walden et al., 2018), this URM label tends to be the most popular in naming WOC and describing the target audience for mentoring STEM efforts. Using this term to represent all WOC overshadows the ways people are subject to inequities (Guy & Boards, 2019) born from mutually constitutive oppressive acts. Additionally, “it is necessary to take into account the social, cultural, and historical context of exclusion, discrimination, and educational apartheid as experienced by fully formed racial and ethnic groups, rather than abbreviated URMs” (Bensimon, 2016, p. 5).

In the rare instances when “underrepresented” is replaced with another label, attention to intragroup difference remains largely ignored. For instance, mentoring for gender equality tends to focus on the basic idea of a woman, not the multiple expressions of womanhood (Thomas et al., 2015). The Katz et al. (2017) examination of Smith College’s Achieving Excellence in Mathematics, Engineering, and Science (AEMES) scholars program highlights the outcomes for undergraduate WOC. Notably, in a study on creating inclusive learning environments to excel, the criteria for WOC implied traditional gender norms. We concur with Chambers et al. (2016) and Ireland et al. (2018) that the literature fails to capture the multidimensional nature of social identity. As a result, little is known regarding how African American/Black, Native American, Latina, Asian American and/or Pacific Islander female STEM students make sense of mentoring efforts.

The URM label overshadows WOC as well as the uniqueness of their social contexts. Furthering the use of noncritical frameworks (i.e., STEM camps) to interpret barriers, such as the lack of STEM identity development for WOC, encourages decontextualized strategies. Our research noted STEM mentoring models with no identified empirical research on specific STEM disciplines. Instead, research suggests that one mentoring model can be applied to all STEM ranks (faculty mentors, peer mentors). Since Asian American women tend to be overrepresented in certain sciences (Fry et al., 2021), assuming a one-size-fits-all approach for STEM disciplines is shortsighted. Discussed in the next theme, the universalism of such actions remains tethered to deficit thought.

### Fix the URMS

For underrepresented students/women of color, phrases such as “low self-efficacy”, “unskilled”, and “outsider perspectives” becomes social factors identified as a hindrances toward STEM success (Brown et al., 2018; Carver et al., 2017; Haegar & Fresquez, 2016; Scott et al., 2017). Likewise, common mentoring model responses to these presumed deficiencies focus on teaching WOC coping strategies that mimic dominant behaviors (Brown et al., 2018; Carver et al., 2017). Efforts to enhance belonging in STEM—such as modeling scientific behavior, teaching study skills, and setting expectations to plan course schedules (Cavnar & Stanny, 2018; Thiry & Laursen, 2011)—proffer behavior-based change. In the work of Blake et al. (2013), dominant themes of assimilation and socialization resulted in mentoring strategies aimed at enhancing interest and capacity in STEM. Our search did not produce any studies in which faculty mentors encouraged mentees to critique systems of oppression.

We found some evidence of near-peer mentoring that, at the least, recognized barriers, even if there was limited reassurance to change them. Zaniewski and Reinholz (2016) describe how a near-peer mentoring model to increase STEM success for underrepresented minorities (defined as African American, American Indians/Alaska Natives, and Latino) assumed a connectedness culture of relationality philosophy. The authors explained that the near-peer mentor implementation process “normalized and empathized with the experiences of their mentees”; consequently, students became part of a community (p. 10). Herein, a mentor/mentee coalition formed between the undergraduate and graduate students that resulted in positive outcomes—belonging, identity formation, persistence, and retention for underrepresented groups in STEM education (Wilson et al., 2012; Zaniewski & Reinholz, 2016). While the near-peer model for Zaniewski and Reinholz philosophically positioned the underrepresented students to cope with forms of oppression using psychosocial and academic support, intersectionality did not inform these processes for mentorship.

Few mentoring models recognize race, gender, racism, sexism, or intersecting identities. Two exceptions are explorations of faculty–student interactions by Griffin et al. (2010) and McCoy et al. (2015). Faculty mentoring has been a tool used to respond to the underrepresentation of specific student groups in STEM HE. Of studies reviewed on faculty mentoring, racialized and gendered disparities seemingly influenced student perceptions about mentoring (Kendricks et al., 2013) as well as the methods employed in mentoring (McCoy et al., 2015). The underrepresentation in STEM HE catalyzed inquiries into the mentoring of students of color by Black and White faculty members.

In two separate studies, Griffin et al. (2010) and McCoy et al. (2015) explored how faculty engaged in mentoring underrepresented students. Though both studies focused on issues of race and racism, the objectives depended upon the faculty mentor’s race. Black faculty mentors, according to a study by Griffin et al (2010), prepared students of color to cope with the unavoidable realities of STEM inequities. They contended that for Black faculty, mentoring students of color was an act of giving back. However, a study by McCoy et al. (2015) offered an alternative perspective on White faculty mentoring students of color. Although White faculty members mentored White, Black, and Brown students at a Historically Black College or Universities (HBCU) and a Predominantly White Institutions (PWI), the mentors did not adjust their styles or strategies. The social contexts in which the students navigated the HE contexts and the ecological impacts on STEM identity or experiences were not considered.

While this study is needed, it examined only race but not race and gender, which has contributed to the paucity of literature in STEM using intersectionality related to the experiences of WOC. Likewise, the application of a critical framework (intersectionality in the approach to mentoring) did not appear in the study; using this critical framework might have led to a different outcome. This example of colorblind and gender-blind mentoring problematized the role of faculty mentoring’s impact on encouraging the representation of racial and gender diversity in STEM HE. Because faculty mentors in STEM higher education influence URM actions, research elevates mentoring’s status as an effective strategy. In some cases, mentors advance potential outcomes to seek resolutions that resist inequities (Griffin et al., 2010) or concede to the dominant culture and accept that nothing can be done to end inequities (Kendricks et al., 2013). The embedding of mentoring within an intervention, such as undergraduate research experiences to increase self-efficacy or content knowledge (Zaniewski & Reinholz, 2016), promotes a narrative that the student, not the system, needs to be fixed (Cavnar & Stanny, 2018; Haegar & Fresquez, 2016; Wilson et al., 2012).

### Branding race–gender or race–gender as a brand

Noticeably, some research (Aikens et al., 2017; Smith et al., 2019) on WOC in STEM uses identity traits (e.g., race and/or gender) to inform efforts aimed at stimulating and/or increasing WOC’s interests. Often, these approaches are shortsighted and examine intersecting identities in a limited capacity. For example, Lisberg et al. (2018) approach URM academic success in STEM using strategies—such as routine study schedules and peer mentoring to build content knowledge—that minimize social issues (i.e., oppression) experienced due to race and gender. Studies with an independent focus on race or gender (Chang et al., 2016; McCoy et al., 2015) rather than studies on mentoring in STEM HE that examine race and gender (Aikens et al., 2017; Smith et al., 2019) are more common. From an intersectional perspective, the limited examples that observe both race and gender point to the flaws of mentoring in STEM HE where multiple identity markers are not considered, which further racializes gender inequities (Smith et al., 2019). Similarly to how mentoring in STEM HE serves to support a financial need (i.e., money for tuition) (Ryan et al., 2014), the framing of circumstances often makes gender, race, and class distinctions ahistorical, decontextualized, and unproblematic without connecting these traits with an intersectional lens (Chang et al., 2016; Smith & Wingate, 2016).

Focusing on one or two aspects of a person’s social identity (race or gender) while excluding all others (ability, class, nationality, etc.) opposes relationality as a significant pillar of intersectionality. Additionally, the distinctive experiences of WOC in relation to their social identities are misinterpreted and mistreated (Amaya et al., 2018; Katz et al., 2017). The implications of such neglect result in underdeveloped identities in STEM, low self-efficacy, and feelings of exclusion and othering (see Avolio et al., 2020; Blickenstaff, 2005; Carlone & Johnson, 2007; Espinosa, 2011; Leath & Chavous, 2018; Moore et al., 2020; Wilkins-Yel et al., 2019).

The constraints on mentoring in STEM HE severely impact WOC. Part of the reasons that WOC do not benefit from an intervention to support content knowledge development, psychosocial needs, financial, or research experience is that mentoring models mirror social norms (Blake et al., 2013; Braun et al., 2017). Stated differently, mentoring has functioned for generations as a one-way transfer of knowledge and unequal power dynamics, where historically marginalized groups have not influenced decisions to promote social equity. Therefore, the likelihood of mentoring frameworks tailored to all individuals, specifically WOC, declines when the decisions on what is needed are generalized (Blake et al., 2013; Ryan et al., 2014). Dominant norms persist without an analytical tool to contextual initiatives for social change.

In most cases, subtle misconceptions guide the mentor model design in STEM HE, often undergirding superficial investigations into the causes of underrepresentation. Other times, systemic inequity in STEM necessitates an equity-seeking mentor structure focus with collective impact in STEM HE (Guy & Boards, 2019; Slovacek et al., 2011). Utilizing a multifaceted mentoring technique, Kobulnicky and Dale’s (2016) study to diversify STEM HE demands that individuals repurpose mentoring for underrepresented people. They prescribe a new mentoring framework in which compatibility in the interactions is determined by the mentor/mentee personality traits using a community framework that does not apply an intersectional lens in the mentor model development. Growing literature calling for a paradigmatic shift, such as counterspaces (Ong et al., 2018) and peer groups (Thomas et al., 2015), features a sociopolitical turn from complacency to advocacy in which issues related to race and/or gender inequity incite the call for change.

Nontraditional methods of mentoring (see Kobulnicky & Dale, 2016) accompany efforts to motivate, support, and/or affirm WOC in STEM HE that illuminate the strength of diversity (Mondisa, 2018). The shared consensus that diversity has been missing spurred methodological changes to mentor models in STEM HE to bolster participation of underrepresented students. To this point, Byars-Winston et al. (2018) explore diversifying the sociocultural scientific environment using cultural-awareness mentoring strategies that broaden the scope of analysis to examine varying identity traits that influence the way people experience life. The need for diversity of thought and action guides the work to create new goals of mentoring for equity in STEM HE at collective and institutional levels.

While the literature speaks to the frequent use of mentoring to improve examples of high attrition and feelings of isolation among underrepresented groups in STEM HE (Gross et al., 2015), diversity and inclusion strategies for mentoring are infrequent (Smith & Wingate, 2016). WOC are least impacted by mentoring reforms because efforts emphasize survival and coping mechanisms to enact change in STEM HE (Ghee et al., 2016). Establishing a framework where WOC are included as unique and equal while being grouped with those deemed underrepresented socially and/or culturally in STEM HE continues. As long as mentoring programs that emphasize improving gender or race representation in STEM HE assume interventions where no critical lens (intersectionality) informs the scope of the work, WOC will gain little to nothing.

Although inequity dominates the need for mentoring in STEM HE, most actions to understand and implement mentoring appear shortsighted (McCoy et al., 2015). Overwhelmingly, STEM HE studies on mentoring provide few examples that explore systemic inequities through a cultural lens with multiple identity markers (Kobulnicky & Dale, 2016). Likewise, foreseeable changes often suggest ongoing uniform practices of reifying differences in order to marginalize and not recognize diversity as a valuable attribute (Carroll & Barnes, 2015) that guides the role of mentoring WOC.

In general, the results of our first research question (What impact do the social contexts of WOC have on their mentoring experiences in STEM HE?) reveal that despite efforts to “upskill” WOC in STEM, the HE STEM mentoring models consist of saturated sites of violence—that is, contexts in which the knowledge of HE remains uncontested and perpetuates dominant narratives of WOC. Without interrogating why HE mentoring models include the above deficit strategies, the “acceptable knowledge [of STEM HE mentoring models] reflects and protects the dominant group’s status of power and privilege, otherwise known as *epistemic oppression*” (Rankin et al., 2021, p. 26:2). Without providing meaningful opportunities for WOC to narrate how they navigate oppressive systems, the social context maintains epistemic violence. Much like in computer science education, HE STEM models support a race–ethnicity–gender blind spot that refuses to acknowledge these distinctions as “contributing factor(s) to the differential treatment” that WOC experience (ibid).

### General observations of the current state of the literature

Importantly, we note how intersectionality has been positioned on behalf of WOC in STEM to problematize the normative HE cultures. Seminal work has explored the marginalization of WOC and the barriers to their persistence and participation in STEM (Charleston et al., 2014; Espinosa, 2011; Guy & Boards, 2019). Studies have embraced intersectionality and revealed the psychological processes and educational outcomes of the disenfranchised locations of WOC (Charleston et al., 2014; Ireland, et al., 2018), and they have documented their resistance to such processes of exclusion and isolation (Ong et al., 2018).

Social factors, such as devaluing Indigenous knowledge practices and excluding WOC as participants and experts in the field (Guy & Boards, 2019; Wilkins-Yel et al., 2019), have become the norm (Slovacek et al., 2011). Since the 1976 landmark report “The double bind: the price of being a minority woman in science” (see Malcom et al., 1976), institutions of HE have been encouraged to recognize and ameliorate these oppressive practices. Mentoring models are attempts to address the consequences of othering WOC students in STEM.

While scholars have put forth substitutive efforts to address the marginalization of female students of color, disparities continue (Malcom & Malcom, 2011; The National Academies of Sciences Engineering Medicine, 2018). From 1976 to the present, the need for systemic change persists. Yet little is known regarding which and how social contexts affect mentoring models for female STEM students of color. For example, the exploration of Smith et al. (2019) of intersecting identities’ (i.e., race, gender) influence on Black women’s construction of intersecting STEM identities noted themes of “high private regard” and “low public regard” as contributors to their STEM success. Equally important, the use of intersectionality by Ong et al. (2018) to examine the experiences of women of color in STEM provides insight that reflects the complexity of their lived experiences. While these studies broaden understanding and build capacity beyond teacher and counselor education programs, both apply intersectionality to reveal how different groups of WOC survive and redefine their identities in the absence of intentional efforts. What then happens to identity markers when strategic models to improve achievement and HE experiences (e.g., mentoring models) are offered?

The incremental growth of literature on intersectionality in STEM education has raised questions on both the style of mentoring in respect to diverse populations and the influence diversity has on understanding and practices of mentoring. While some studies on underrepresented groups disaggregate participant data by factors such as gender and race/ethnicity, most do not. Some may argue that disaggregating data may be unethical because it exposes the minuscule underrepresented participant’s identity. Regardless of the small numbers, the relevance of applying an intersectional framework to mentoring models does not change. According to Ireland et al. (2018), significant work needs to be done to center intersectionality as a theoretical and methodological framework in educational research. The benefits of doing this would include positioning education researchers to expand reform critiques into the sense of some researchers’ attempts to theorize, integrate, and bring attention to the benefits of intersectionality in STEM HE. Theoretically and methodologically, there exists a paucity of research on mentoring that uses an intersectional framework. Thus, it is necessary to advance an approach that considers the total experience for WOC to decrease attrition or lack of enrollment, increase retention, and enhance the academic experiences, outcomes, and success for WOC in STEM HE.

Near-peer and faculty mentors represented most mentoring strategies that explore, interpret, or recognize effective mentoring relationships in STEM HE in comparison to community-based (Kobulnicky & Dale, 2016) and cultural-awareness mentor models (Byars-Winston et al., 2018). Under the same objective to ameliorate systemic inequity from social disparity, near-peer and faculty mentor models operate from different perspectives that do not offer explicit or implicit considerations of intersectionality as a construct in the mentoring framework. The following paragraphs briefly describe the near-peer and faculty mentor models’ philosophical backgrounds and implementation processes as described in the studies reviewed.

In taking a direct approach to mentoring as a two-way process between participants, Tenenbaum et al. (2014) explored the experiences of near-peer mentors in a summer program. Based on the authors’ espoused philosophy of mentorship encouraging personal development, their study’s implementation of a near-peer mentor model included trainers and activities. The study found benefits from a near-peer mentor model that uses inquiry-based STEM modules, educational and career guidance, and relationship building for support. The observation of diversity by race and gender are recorded in the study design where the information appears significant to the study context description, yet insignificant in the training of mentors. In addition, the study values building supportive relationships with mentees from a diverse population, but does not suggest differentiation strategies in the preparation of near-peer mentors.

The investment of Tenenbaum et al. (2014) in preparing a network of underrepresented STEM mentors is also reflected in the study by Trujillo et al. (2015). While both studies emphasize peer collaboration, Trujillo et al. (2015) distinctly highlight the importance of teacher and learner engagement in STEM. Using a teacher/learner near-peer mentor model helps to level the distribution of power. Some interventions to increase retention among traditionally underrepresented students explore options to demystify the research experience with undergraduate research opportunities. However, solutions to address the lack of experiences in research for the underrepresented students translate into mentorship remedies absent of intersectionality as an analytical tool. To this point, Tenenbaum et al. (2014) and Trujillo et al. (2015) suggest that underrepresentation in STEM HE resulted from educator/learner disregard for social disparities’ impact on self-efficacy, belongingness, and professionalism without examining them with an intersectional lens.

Examples of faculty mentors are also used to improve the presence of underrepresented groups in STEM HE. Faculty mentoring has become a tool to respond to the underrepresentation of specific student groups in STEM HE. Of studies reviewed on faculty mentoring, racialized and gendered disparities seemingly influenced student perceptions of mentoring (Kendricks et al., 2013) as well as the methods employed in mentoring (McCoy et al., 2015). Informal and formal faculty mentoring in STEM education became performative to the culture of HE. Faculty mentoring was characterized as the cultivator of social change (Griffin et al., 2010); this philosophy undergirded most efforts to implement models that disrupt systemic forms of inequities’ influence on minority student performance in STEM (Kendricks et al., 2013).

According to Kendricks et al. (2011), an “othermothering” style of mentoring, in which faculty mentors served as extended family (mothers, fathers, sisters, etc.), reported higher rates of retention and academic achievement among minority students. Kendricks et al. further described how the HBCU study context approach to improve student retention rates uses socialization tactics in STEM HE that are familiar to students’ lived experiences and draws on assets-based ideologies. Thus, faculty mentoring in STEM higher education for underrepresented students became less about content mastery and more about successfully navigating cultural spaces that are atypical of the dominant norm (Griffin et al., 2010; Hayes & Bigler, 2013). These instances of customizing mentorship in STEM HE indirectly illustrated how intersectionality can facilitate ideal mentoring experiences when the faculty mentor interactions are informed by multiple identity markers that define an individual (Smith et al., 2019).

The underrepresentation of racially and ethnically marginalized students in STEM HE catalyzed inquiries into mentoring students of color by Black and White faculty members. In two separate studies, Griffin et al. (2010) and McCoy et al. (2015) explored how faculty engaged underrepresented students while mentoring them. Although faculty study participants focused on issues of race and racism, the objectives sharply differed between the Black and White faculty. Our review suggests the following two primary reasons motivate the creation of mentoring models for WOC: (1) to provide WOC strategies to navigate unfamiliar and often hostile STEM HE environments (Graham, 2019; Grant & Ghee, 2015); and (2) to cultivate a greater sense of belongingness and science self-identity in normative STEM cultures (Kendricks et al., 2013; Ong et al., 2018; Zaniewski & Reinholz, 2016). We did not identify any literature illustrating how mentoring models contribute to the complexity of identities of WOC and their positionings in the web of social and structural relations while navigating HE. Rather, the reported outcomes of mentoring WOC show how they effectively (or not) overcome barriers and negative experiences (Espinosa, 2011). Generally, critical analysis of mentoring models for WOC in STEM fail to offer specific recommendations. Thus, the next section provides a partial list of specific suggestions for constructing and revising mentoring models for WOC.

### Conceptualizing intersectionality in STEM mentoring: discussion and recommendations

In using intersectionality as a theoretical frame and analytic lens for this paper, we intended to highlight the ways in which the framework has been overlooked in discussions of mentoring for WOC in STEM HE. Evident from our analyses of literature to answer research question one (What impact do the social contexts of WOC have on their mentoring experiences in STEM HE?) is the establishing of mentor models that serve the needs of White, Western, upper/middle-class, able-bodied, male students and not students of color, women, differently able-bodied individuals, etc. Essentially, we found that WOC’s social contexts did not influence the design of mentor models toward more inclusive practices. In answering our second and third research questions (What role does intersectionality play in the structural organization of WOC mentoring models in STEM HE? and How has intersectionality shaped WOC mentors’ and mentees’ life experiences?) our literature synthesis revealed that intersectionality is conspicuously absent from mentoring models focused on WOC in STEM HE. Social context, relationality, and power are not taken into account despite the opportunity to include and validate the experiences of WOC. Consequently, this following section responds to our fourth research question (How can mentoring models utilize intersectionality to incorporate the experiences of WOC in STEM HE?).

Ultimately, we argue that mentorship is incomplete if not considered through an intersectional lens. In keeping with this central argument and the findings discussed above, we offer a set of guiding principles for transformational mentoring practices. We understand that our proposed strategies may be perceived as conceptually abstract. In part, the challenge lies in delivering nuanced, intersectionality-informed one-on-one mentoring that has never been done. Such an approach is not generalizable or simple. Intersectionality is about recognizing complexity. As was referenced above, in understanding intragroup difference, which was inevitably shaped by Collins’ (2019) core constructs, mentors and mentees need to work toward collectively analyzing how these constructs lead toward social injustice.

**Fig. 3:**
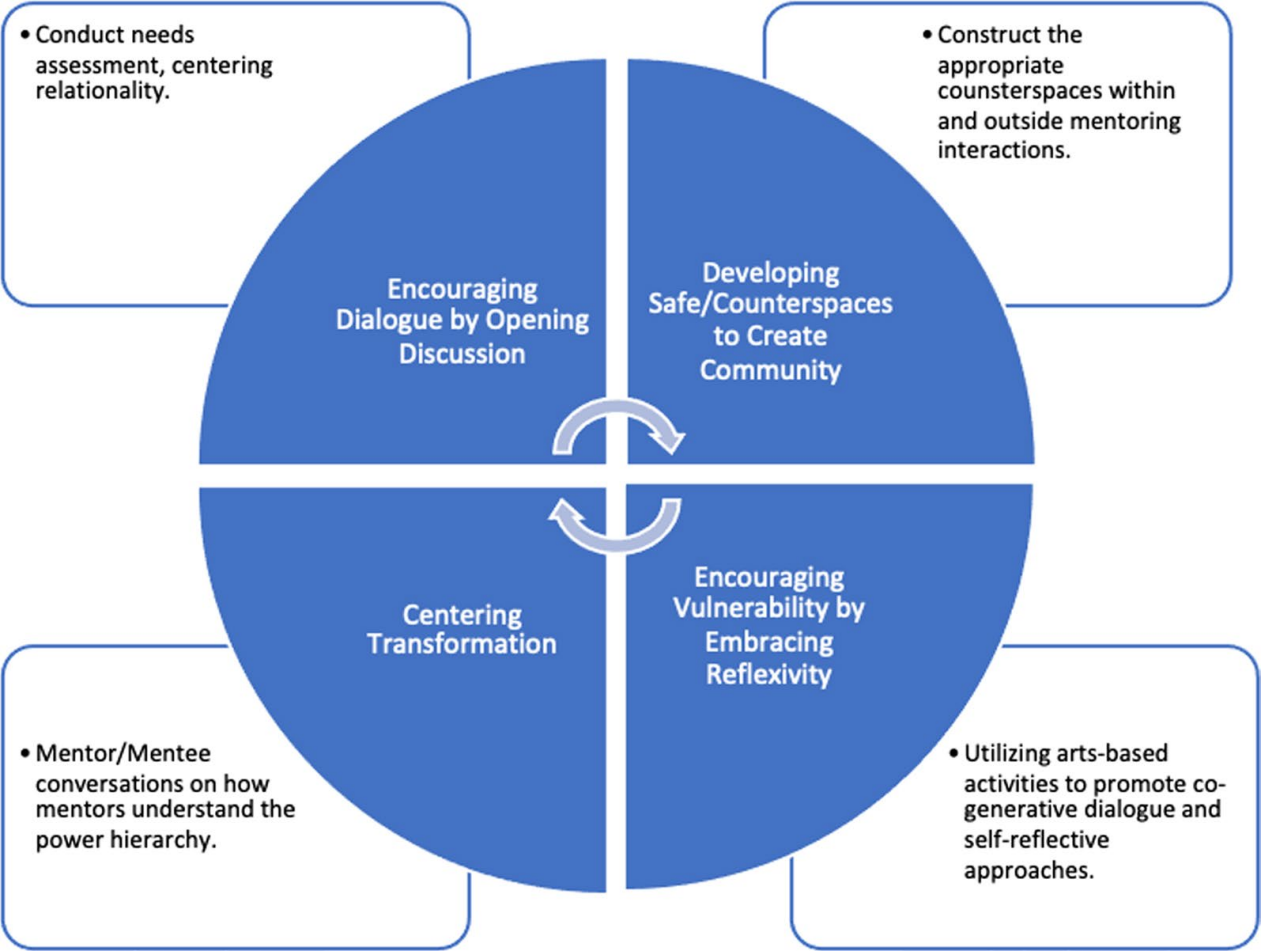
Recommendations for conceptualizing intersectionality in STEM mentoring

Much like the recommendations from Boyce (2021) that were related to constructing appropriate mentoring strategies for students of color, our holistic suggestions include adopting an intersectional lens. These recommendations were formed through a critical read of the existing literature and center the following three aspects that we felt were often missing from previous studies: (1) accounts of the lived experiences of WOC in STEM HE; (2) the validation of their complex interrelated struggles with issues of social context, relationality, and power; and (3) the acknowledgment of their place within broader systems of racism, sexism, classism, and elitism (Fig. 3). Our intent is to offer readers action points and critical questions that can be used in the mentoring process to foster a social justice consciousness in mentors and mentees regarding existing STEM HE systems and their impact on WOC. When combined with an intersectional framework and an understanding of the patterns of existing STEM HE systems, these principles can be used to guide the development of transformational mentoring practices that move us toward more equitable STEM education futures.

### Encouraging dialogue by opening discussion

To engage in truly transformational mentoring practices, an authentic and genuine dialogue about the needs and concerns of WOC in STEM HE is crucial. As a first step, we suggest a needs-assessment activity (i.e., equity framework that includes questions not based on individual needs but what they think the institution should do to support their needs) between the mentor and mentee be performed prior to an in-person meeting. Potential questions to the mentee embedded within the assessment activities are the following: What does a safe space look like in your department and how do you suggest your institution or department create one? What kinds of policies and/or procedures do you believe will assist you in your professional achievements? The same interrogatives should be posed to the mentor. Departments and institutions can sustain these needs-assessment activities by making it a requirement for both mentors and mentees.

Data from these assessments can be used in mentoring to identify and highlight the barriers to the participation, retention, and success of WOC in STEM and to brainstorm potential solutions. Key to this process is the notion of relationality, in which the interconnections between social categories, subject formations, and power structures are recognized as mutually productive and reinforcing. While the idea of establishing a dialogue about the needs and concerns of WOC may seem overly simplistic, a thorough reading of the existing research on mentoring proves this step is too often overlooked or considered an afterthought; it is an evaluation of the mentoring performance after the fact. Making dialogue the first guiding principle, or the first step toward transformational mentoring practices that is maintained throughout the mentoring relationship, ensures its inclusion as a key facet of mentoring design and amplifies the voices of WOC in a direct way.

### Developing safe/counterspaces to create community

One of the most important factors related to the persistence and retention of WOC in STEM HE spaces is the development of a sense of belonging or self-efficacy (Guy & Boards, 2019; Ong et al., 2018). While there are many methods of increasing belonging and science self-efficacy, we believe the creation of STEM safe/counterspaces is one of the most promising because it can be found naturally outside academic structures and formed within them as part of an intentional move toward inclusivity and community development. Ong et al. (2018) found that when STEM departments intentionally served as counterspaces for women of color, they had the potential to “disrupt patterns of privilege and marginalization” (p. 233). It would be challenging to present a standard model of a safe space; however, we suggest using results from the needs assessment to construct the appropriate counterspace for mentees. In general, the literature indicates that providing opportunities for growth and affirmation cultivate a strong sense of self, which is a significant element constituting a counterspace. How to specifically operationalize these elements should be based, at least in part, on mentees’ perspectives.

In transformational mentoring practices, the development of safe and/or counterspaces is to be prioritized in order to create community among WOC in traditionally hostile STEM HE environments. These communities can serve any number of functions, from supporting the development of social and interpersonal skills for advancement, to helping WOC build the various forms of intellectual and cultural capital needed to establish their place within STEM HE, to easing some of the personal and psychological burdens traditional STEM structures place on their shoulders. These environments also should be places for authentic dialogue, as outlined above, and should operate within and outside of mentoring interactions. This guiding action of establishing STEM departmental counterspaces may require more institutional support than other programs, but it has become increasingly necessary as a way for STEM departments and institutional academic structures to signal their willingness to support and nurture WOC students. The development and continued support of STEM safe/counterspaces provides one way for STEM departments to facilitate the academic success of their WOC students while simultaneously serving their personal, social, and emotional needs. To this end, STEM departments may do well by actively laying bare how power operates.

### Encouraging vulnerability by embracing reflexivity

Defined in feminist research as the practice of “attempting to make explicit the power relations and the exercise of power in the research process”, reflexivity can be used in multiple contexts to unpack the relation of politics and epistemology with knowledge production, power relations, and social positioning (Ramazanoglu & Holland, 2002, p. 118). As a tool for critical reflection, reflexivity is important in helping researchers uncover “the exercise of power, power relationships, and their effects in the research process” while holding researchers accountable for the knowledge produced (Ramazanoglu & Holland, 2002, p. 119). Importantly, reflexivity is an “invitation to other voices to challenge the researcher’s knowledge claims and concepts of power” and is both a collective and contested practice, opening possibilities for critical dialogue and negotiation (Ramazanoglu & Holland, 2002, p. 119).

In transformational mentoring, reflexivity should be embraced as an essential component of STEM learning and engagement. In mentoring, reflexivity encourages the development of critical reflection skills for mentors and mentees, inviting vulnerability into mentoring interactions by asking participants to seriously consider their relationship to power, ethical judgments, and notions of accountability. Nontraditional ways of encouraging both mentors and mentees to embrace reflexivity include integrating arts-based activities, specifically, using theater of the oppressed methods of gaming activities, image theater, and/or forum theater to build trust and an appreciation of how to share power (Agosto et al., 2022). These activities use a self-reflective approach that connects to the needs assessment by allowing mentors and mentees to engage in co-generative dialogue on how to form supportive and productive relationships. Reflexivity also creates space for mentors and mentees to be wrong and have their positions challenged and their beliefs contested as part of a collaborative or reciprocal knowledge-sharing process. Further examples of how to emphasize reflexivity (Fox & Leeder, 2018) in mentoring range from considering the identities of mentoring participants and how they play a role in shaping interpersonal, structural, and disciplinary power relations to the contextualizing of mentoring and STEM teaching/learning within normative STEM HE cultures (i.e., catering to primarily White male able-bodied students and teachers). To illustrate, Boal’s (2002) image theater focuses on the articulation of thought using the body to express realities specific to the individual. This exercise invites the mentor and mentee to reflect on nonverbal methods around topics that are connected to experiences, understandings, and interpretations of mentoring relationships. While reflexivity may seem like an untenable vulnerability—especially in mentoring interactions, which are traditionally hierarchical—it is an important aspect of transformational mentoring because of its ability to encourage deeper reflection on the role of power in the mentoring process and to produce more equitable patterns of relating across participants.

### Centering transformation

The most important (and most difficult) of the four guiding principles is centering transformation, or the practice of mentoring for social transformation. Socially transformative mentoring works through centering social justice concerns and equitable patterns of relating in every part of the mentoring process. No part of STEM learning occurs in a vacuum, and pretending that cultural norms and social values do not influence STEM learning and mentoring is akin to “sticking one’s head in the sand”. To solely focus on the academic or professional development elements necessary to advance students through STEM HE or to emphasize the socialization or assimilation of WOC students into dominant STEM norms is to leave aspects of the lived experiences WOC unrecognized and unrealized. It is a failure to engage mentoring with an intersectional frame that implicitly devalues the WOC who participate in mentoring relationships. Rather, conversations between mentors and mentees should include how mentors understand the power hierarchy in their specific context; ways they have navigated it along various lines of identity (e.g., race, gender, sexual orientation); and potential strategies to transform the system using an equity lens. As mentors bring attention to unequal power dynamics, reflect on their sustainability, and co-construct strategies to upend policies and procedures, they will create the “paradigm shift” that Collins (2019) encourages in intersectional work. This suggestion reflects a project-based design that aims to deconstruct the failing STEM HE system toward more equitable HE futures for all.

While the principles outlined above have started the process of centering social justice concerns and establishing equitable relations, we also offer a list of questions for mentors and mentees to consider in moving toward transformational mentoring practices (see Additional file 1: Appendix A). We suggest that questions specific to a mentor and a mentee should be completed before/during/after a mentoring engagement. Such interrogatives are intended to serve as a guide for mentors, teachers, and other education practitioners who wish to center the principles of intersectionality and social justice but lack a clear idea of how to do so. While the majority of this paper advances a structural analysis of relationality, power, and social context in STEM HE practices, these questions are a way to practically implement an intersectional framework in daily mentoring practices. Thus, these questions take an interpersonal or individual approach to addressing the disparities of STEM HE. Change on the individual level alone cannot solve the problems of an inequitable HE system, but it is a start that, in time, can be scaled to address structural problems across HE administrations and STEM cultures.

This list of questions and the guiding principles outlined above are neither exhaustive nor prescriptive. Instead, they are intended to be altered and expanded upon to support the development of a social justice consciousness for STEM HE mentors and mentees. These questions and principles should be used in future research studies to solicit feedback from the WOC in STEM HE to help identify what makes a good mentor, what may contribute to mentee perceptions of good mentorship, and what mentoring practices are the most equitable and impactful for whom.

It would be impossible to craft a mentoring program that caters to multiple intersections. Manifold approaches specific to any one group will inevitably leave out important intersecting identities among other groups and their lived experiences. Our suggestion is the following: more attention needs to be paid to establishing a mentoring program that understands power, challenges social context, and interrogates oppressive structures. Mentors and mentees, as well as other individuals in higher education (e.g., staff and administrators) need a platform to address hierarchies. Space needs to be provided in which WOC are empowered to challenge and transform interconnected power structures. Is this a one-size-fits-all approach? To some extent, it is; but this approach is also one that is open and non-constraining to individual needs.

## Conclusion

If the tenor of our current times has taught us anything, it is that established practices and ways of being in the world are untenable. Inequities that make up the foundation of the U.S. education system have been exposed by the COVID-19 pandemic, from disparate access to reliable internet and distance learning technologies to financial insecurity caused by the lack of access to on-campus jobs and housing (Patel, 2020). The only way to solve these problems is to seek out and value the voices and experiences of those who live through, work in, and resist oppressions as well as let their insight guide our approach to repairing our HE systems. Starting with STEM education and mentoring approaches, we argue for a centering of WOC and intersectionality in the mentoring process as a way of not just repairing our HE systems, but also building better, more equitable ones.

Notable study limitations within our search criteria highlight a lack of specific disciplinary categories (as defined by the National Science Foundation (NSF) definition of STEM along with medicine), including disciplinary categories of science, technology, engineering, math, astronomy, chemistry, computer science, geoscience, life science, physics, psychology, social science, STEM education, and medicine. In future studies, we recommend adding that level of specificity by disciplines rather than the general reference to STEM as a keyword.

Until we view WOC as distinct from the socially constructed, and often stereotypical, version that influences decisions on what works for that group, WOC will continue to lag in enrollment numbers and graduation rates in STEM HE. The mistakes of past efforts to attain social equity for WOC follow the same template of: (1) choosing to minimize their unique experiences with prescribed protocols for research that operate from dominant social norms and (2) discussions that present mentoring as a neutral exercise that is transparent and liberating to the mentee. WOC’s narratives as underrepresented in STEM HE contributes to the impression that they, as a group, have the same needs or will positively respond to mentoring as an intervention. Multiple examples in the literature confirm the role of intersectionality, if considered, to serve to justify the application of nontraditional mentoring strategies, therefore making an intersectionality lens less analytical and more descriptive. Moreover, where intersectionality as an analytical lens could expose the flaws in mentor models designed for women and people of color, the omission of intersectionality in the mentoring framework furthers a reliance on dominant norms. Intersectionality has a place in mentoring, especially for contextualizing the significance of diversity when examining the impact on WOC with different lived experiences. Because some studies categorize mentoring for WOC as a resistance knowledge project, this means “the guiding question is less whether to resist prevailing power arrangements and more what forms such resistance might take” (Collins, 2019, p. 116). Yet the question remains: *How does the resistance knowledge project specifically relate to WOC and why*?

## Supplementary Information

**Additional file 1.** NCES/IPEDS—graduation/completion rates over time.

## Data Availability

The articles used for this study represent the example data and are available to all researchers. The references indicate which articles were analyzed through the cited study. See Additional file 1: Appendix for a complete list of studies included.

## Abbreviations

STEM: Science, technology, engineering, and math
HE: Higher education
URM: Underrepresented minority
WOC: Women of color
AEMES: Achieving excellence in mathematics, engineering, and science
HBCU: Historically Black College or University
PWI: Predominantly white institution
NSF: National Science Foundation

## Footnotes

1 We understand that the terms Hispanic and Latina are not used interchangeably by individuals belonging to either of these ethnic groups. However, these labels are applied as synonyms in most of the research and reports we reviewed.

2 Importantly, these words do not represent one monolithic group. However, most non-Indigenous research lumps these communities together using one of these three terms to represent individuals belonging to any one of these social categories.

3 Degrees are considered “completed” if students completed their degree/certification program within 150% of “normal time” (assumed to be the 4-year completion rate of first-year undergraduates at a public university).
