# Supplementary material for: Mentoring in STEM higher education: a synthesis of the literature to (re)present the excluded women of color
Source: Int J STEM Educ. 2022 Jul 29;9(1):50. doi: 10.1186/s40594-022-00367-7 (PMC9336123; doi:10.1186/s40594-022-00367-7)
Supplement: Supplementary file 1 — Additional file 1. NCES/IPEDS—graduation/completion rates over time. [file 40594_2022_367_MOESM1_ESM.doc]

Appendix

Articles included in the study

| # | Article |
| --- | --- |
| 1 | Aikens, M. L., Robertson, M. M., Sadselia, S., Watkins, K., Evans, M., Runyon, C. R., Dolan, E. L. (2017). Race and gender differences in undergraduate research mentoring structures and research outcomes. *CBE—Life Sciences Education*, *16*(2), 1–12. <http://dx.doi.org.ezproxy1.lib.asu.edu/10.1187/cbe.16-07-0211>. |
| 2 | Amaya, L. R., Betancourt, T., Collins, K. H., Hinojosa, O., & Corona, C. (2018). Undergraduate research experiences: Mentoring, awareness, and perceptions—a case study at a Hispanic-serving institution. *International Journal of STEM Education*, *5*(1), 1–13. <https://dx.doi.org/10.1186/s40594-018-0105-8>. |
| 3 | Blake, R. A., Liou-Mark, J., & Chukuigwe, C. (2013). An effective model for enhancing underrepresented minority participation and success in geoscience undergraduate research. *Journal of Geoscience Education*, *61*(4), 405–414.  DOI: <https://dx.doi.org/10.5408/12-417.1>. |
| 4 | Bosman, L., Chelberg, K., & Winn, R. (2017). How does service-learning increase and sustain interest in engineering education for underrepresented pre-engineering college students? *Journal of STEM Education: Innovations and Research*, *18*(2). |
| 5 | Braun, D. C., Gormally, C., & Clark, M. D. (2017). The deaf mentoring survey: A community cultural wealth framework for measuring mentoring effectiveness with underrepresented students. *CBE—Life Sciences Education*, *16*(1), ar10. [https://dx.doi.org/10.1187/cbe.15-07-0155](https://doi.org/10.1187/cbe.15-07-0155). |
| 6 | Brown, O., Morris, M., Hensel, R., & Dygert, J. (2018, June). An integrated supplemental program to enhance the first-year engineering experience. In *ASEE Annual Conference Proceedings*. |
| 7 | Byars-Winston, A., Womack, V. Y., Butz, A. R., McGee, R., Quinn, S. C., Utzerath, E., Saetermoe, C. L., & Thomas, S. B. (2018). Pilot study of an intervention to increase cultural awareness in research mentoring: implications for diversifying the scientific workforce. *Journal of Clinical and Translational Science*, *2*(2), 86–94. <https://dx.doi.org/10.1017/cts.2018.25>. |
| 8 | Carroll, M. A., & Barnes, E. F. (2015). Strategies for enhancing diverse mentoring relationships in STEM fields. *International Journal of Evidence Based Coaching and Mentoring*, *13*(1), 58–69. |
| 9 | Carver, S. D., Van Sickle, J., Holcomb, J. P., Jackson, D. K., Resnick, A., Duffy, S. F., Sridhar, N., & Quinn, C. M. (2017). Operation STEM: increasing success and improving retention among mathematically underprepared students in STEM. *Journal of STEM Education: Innovations and Research*, *18*(3), 20. |
| 10 | Carpi, A., Ronan, D. M., Falconer, H. M., & Lents, N. H. (2017). Cultivating minority scientists: undergraduate research increases self‐efficacy and career ambitions for underrepresented students in STEM. *Journal of Research in Science Teaching*, *54*(2), 169-194. <https://dx.doi.org/10.1002/tea.21341>. |
| 11 | Chang, J. M., Kwon, C., Stevens, L., & Buonora, P. (2016). Strategies to recruit and retain students in physical science and mathematics on a diverse college campus. *Journal of College Science Teaching*, *45*(3), n3. |
| 12 | D’Souza, M. J., Shuman, K. E., Wentzien, D. E., & Roeske, K. P. (2018). Working with the Wesley College Cannon Scholar Program: improving retention, persistence, and success. *Journal of STEM Education: Innovations and Research*, *19*(1), 31–40. |
| 13 | DiBartolo, P. M., Gregg-Jolly, L., Gross, D., Manduca, C. A., Iverson, E., Cooke III, D. B., Davis, G. K., Davidson, C., Hertz, P., Hibbard, L., Ireland, S. K., Mader, C., Pai, A., Raps, S., Siwicki, K., & Swartz, J. (2016). Principles and practices fostering inclusive excellence: lessons from the Howard Hughes Medical Institute’s Capstone Institutions. *CBE—Life Sciences Education*, *15*(3), ar44. <https://dx.doi.org/10.1187/cbe.16-01-0028>. |
| 14 | Estrada, M., Hernandez, P. R., & Schultz, P. W. (2018). A longitudinal study of how quality mentorship and research experience integrate underrepresented minorities into STEM careers. *CBE—Life Sciences Education*, *17*(1), ar9. <https://dx.doi.org/10.1187/cbe.17-04-0066>. |
| 15 | Eubanks-Turner, C., Beaulieu, P., & Pal, N. (2018). Smooth transition for advancement to graduate education (STAGE) for underrepresented groups in the mathematical sciences pilot project: broadening participation through mentoring. *PRIMUS*, *28*(2), 97–117. <https://dx.doi.org/10.1080/10511970.2017.1295409>. |
| 16 | Ghee, M., Keels, M., Collins, D., Neal-Spence, C., & Baker, E. (2016). Fine-tuning summer research programs to promote underrepresented students’ persistence in the STEM pathway. *CBE—Life Sciences Education*, *15*(3), ar28. <https://dx.doi.org/10.1187/cbe.16-01-0046>. |
| 17 | Goonewardene, A. U., Offutt, C. A., Whitling, J., & Woodhouse, D. (2016). An interdisciplinary approach to success for underrepresented students in STEM. *Journal of College Science Teaching, 45*(4), 59–57. |
| 18 | Griffin, K. A., Perez, D., Holmes, A. P., & Mayo, C. E. (2010). Investing in the future: the importance of faculty mentoring in the development of students of color in STEM. *New Directions for Institutional Research*, *2010*(148), 95–103. <https://dx.doi.org/10.1002/ir.365>. |
| 19 | Griffin, K. A., & Reddick, R. J. (2011). Surveillance and sacrifice: gender differences in the mentoring patterns of Black professors at predominantly White research universities. *American Educational Research Journal*, *48*(5), 1032–1057. [https://dx.doi.org/10.3102/0002831211405025](https://dx.doi.org/10.3102%2F0002831211405025). |
| 20 | Gross, D., Iverson, E., Willett, G., & Manduca, C. (2015). Broadening access to science with support for the whole student in a residential liberal arts college environment. *Journal of College Science Teaching*, *44*(4), 99–107.  <https://www.jstor.org/stable/43631871>. |
| 21 | Guy, B., & Boards, A. (2019). A seat at the table: exploring the experiences of underrepresented minority women in STEM graduate programs. *Journal of Prevention & Intervention in the Community*, *47*(4), 354–365. <https://dx.doi.org/10.1080/10852352.2019.1617383>. |
| 22 | Haeger, H., & Fresquez, C. (2016). Mentoring for inclusion: the impact of mentoring on undergraduate researchers in the sciences. *CBE—Life Sciences Education*, *15*(3), ar36. <https://dx.doi.org/10.1187/cbe.16-01-0016>. |
| 23 | Hayes, A., & Bigler, R. (2013). Gender-related values, perceptions of discrimination, and mentoring in STEM graduate training. *International Journal of Gender, Science and Technology*, *5*(3), 254–280. |
| 24 | Hernandez, P. R., Estrada, M., Woodcock, A., & Schultz, P. W. (2017). Protégé perceptions of high mentorship quality depend on shared values more than on demographic match. *The Journal of Experimental Education*, *85*(3), 450–468. <https://dx.doi.org/10.1080/00220973.2016.1246405>. |
| 25 | Katz, L. A., Aloisio, K. M., Horton, N. J., Ly, M., Pruss, S., Queeney, K., Rowen, C. & DiBartolo, P. M. (2017). A program aimed toward inclusive excellence for underrepresented undergraduate women in the sciences. *CBE—Life Sciences Education*, *16*(1), ar11. <https://dx.doi.org/10.1187/cbe.16-01-0029>. |
| 26 | Kendricks, K., & Arment, A. (2011). Adopting a K-12 family model with undergraduate research to enhance STEM persistence and achievement in underrepresented minority students. *Journal of College Science Teaching*, *41*(2). |
| 27 | Kobulnicky, H. A., & Dale, D. A. (2016). A community mentoring model for STEM undergraduate research experiences. *Journal of College Science Teaching*, *45*(6), 17–23. |
| 28 | Lisberg, A., & Woods, B. (2018). Mentorship, mindset and learning strategies: an integrative approach to increasing underrepresented minority student retention in a STEM undergraduate program. *Journal of STEM Education*, *19*(3). <https://www.learntechlib.org/p/184625/>. |
| 29 | Luedke, C. L. (2017). Person first, student second: staff and administrators of color supporting students of color authentically in higher education. *Journal of College Student Development*, *58*(1), 37–52. <http://dx.doi.org/10.1353/csd.2017.0002>. |
| 30 | Luedke, C. L., McCoy, D. L., Winkle-Wagner, R., & Lee-Johnson, J. (2019). Students perspectives on holistic mentoring practices in STEM fields. *JCSCORE*, *5*(1), 33–59. <https://dx.doi.org/10.15763/issn.2642-2387.2019.5.1.33-59>. |
| 31 | MacPhee, D., Farro, S., & Canetto, S. S. (2013). Academic self‐efficacy and performance of underrepresented STEM majors: gender, ethnic, and social class patterns. *Analyses of Social Issues and Public Policy*, *13*(1), 347–369. |
| 32 | McCoy, D. L., Winkle-Wagner, R., & Luedke, C. L. (2015). Colorblind mentoring? Exploring white faculty mentoring of students of color. *Journal of Diversity in Higher Education*, *8*(4), 225. |
| 33 | Mondisa, J. L. (2018). Examining the mentoring approaches of African-American mentors. *Journal of African American Studies*, *22*(4), 293–308. <https://dx.doi.org/10.1007/s12111-018-9411-y>. |
| 34 | Russell, M. L., Escobar, M., Russell, J. A., Robertson, B. K., & Thomas, M. (2018). Promoting pathways to STEM careers for traditionally underrepresented graduate students. *Negro Educational Review*, *69*(1–4), 5–32, 142–143. DOI  10.18260/1-2—19913 |
| 35 | Ryan, R. G., Durdella, N., & Navarro, T. (2014, June). A case study of success: Mentoring and supporting underrepresented transfer students in a mechanical engineering program. In *2014 ASEE Annual Conference & Exposition* (pp. 24–21). <https://dx.doi.org/10.18260/1-2--19913>. |
| 36 | Scott, T. P., Thigpin, S. S., & Bentz, A. O. (2017). Transfer learning community: overcoming transfer shock and increasing retention of mathematics and science majors. *Journal of College Student Retention: Research, Theory & Practice*, *19*(3), 300-316. [https://dx.doi.org/10.1177/1521025115621919](https://dx.doi.org/10.1177%2F1521025115621919) |
| 37 | Schneider, K. R., Bickel, A., & Morrison-Shetlar, A. (2015). Planning and Implementing a Comprehensive Student-Centered Research Program for First-Year STEM Undergraduates. *Journal of College Science Teaching*, *44*(3), 37–43. <http://www.jstor.org/stable/43631937>. |
| 38 | Slovacek, S. P., Whittinghill, J. C., Tucker, S., Rath, K. A., Peterfreund, A. R., Kuehn, G. D., & Reinke, Y. G. (2011). Minority students severely underrepresented in science, technology, engineering, and math. *Journal of STEM Education: Innovations and Research*, *12*(1). |
| 39 | Smith, K.C., Boakye, B., Williams, D., & Fleming, L. (2019). The exploration of how identity intersectionality strengthens STEM identity for Black female undergraduates attending a Historically Black College and University (HBCU). *Journal of Negro Education* *88*(3), 407–418. [https://www.muse.jhu.edu/article/802629](https://muse.jhu.edu/article/802629). |
| 40 | Smith, C., & Wingate, L. (2016). Strategies for broadening participation in advanced technological education programs: Practice and perceptions. *Community College Journal of Research and Practice*, *40*(9), 779–796. <https://dx.doi.org/10.1080/10668926.2015.1108252>. |
| 41 | Tenenbaum, L.S., Anderson, M.K., Jett, M. *et al.* An innovative near-peer mentoring model for undergraduate and secondary students: STEM focus. *Innovative Higher Education* 39**,**375–385 (2014). <https://dx.doi.org/10.1007/s10755-014-9286-3>. |
| 42 | Thiry, H., & Laursen, S. L. (2011). The role of student-advisor interactions in apprenticing undergraduate researchers into a scientific community of practice. *Journal of Science Education and Technology*, *20*(6), 771–784. <https://dx.doi.org/10.1007/s10956-010-9271-2>. |
| 43 | Trujillo, G., Aguinaldo, P. G., Anderson, C., Bustamante, J., Gelsinger, D. R., Pastor, M. J., Wright, J., Márquez-Magaña, L., & Riggs, B. (2015). Near-peer STEM mentoring offers unexpected benefits for mentors from traditionally underrepresented backgrounds. *Perspectives on undergraduate research and mentoring: PURM*, *4*(1). |
| 44 | Wilson, Z. S., Holmes, L., Degravelles, K., Sylvain, M. R., Batiste, L., Johnson, M., McGuire, S. Y., Pang, S. S., & Warner, I. M. (2012). Hierarchical mentoring: a transformative strategy for improving diversity and retention in undergraduate STEM disciplines. *Journal of Science Education and Technology*, *21*(1), 148–156. <https://dx.doi.org/10.1007/s10956-011-9292-5>. |
| 45 | Zaniewski, A. M., & Reinholz, D. (2016). Increasing STEM success: a near-peer mentoring program in the physical sciences. *International Journal of STEM Education*, *3*(1), 14. <https://dx.doi.org/10.1186/s40594-016-0043-2>. |
